# The Differential Effect of Excess Aldosterone on Skeletal Muscle Mass by Sex

**DOI:** 10.3389/fendo.2019.00195

**Published:** 2019-03-29

**Authors:** Mi Kyung Kwak, Seung-Eun Lee, Yoon Young Cho, Sunghwan Suh, Beom-Jun Kim, Kee-Ho Song, Jung-Min Koh, Jae Hyeon Kim, Seung Hun Lee

**Affiliations:** ^1^Division of Endocrinology and Metabolism, Asan Medical Center, University of Ulsan College of Medicine, Seoul, South Korea; ^2^Division of Endocrinology and Metabolism, Department of Internal Medicine, Hallym University Dontan Sacred Heart Hospital, Hwaseong-Si, South Korea; ^3^Division of Endocrinology and Metabolism, Department of Medicine, Samsung Medical Center, Sungkyunkwan University School of Medicine, Seoul, South Korea; ^4^Division of Endocrinology and Metabolism, Department of Medicine, Gyeongsang National University School of Medicine, Jinju, South Korea; ^5^Division of Endocrinology and Metabolism, Department of Internal Medicine, Dong-A University Medical Center, Dong-A University College of Medicine, Busan, South Korea; ^6^Division of Endocrinology and Metabolism, Konkuk University School of Medicine, Konkuk University Medical Center, Seoul, South Korea

**Keywords:** primary aldosteronism, aldosterone, skeletal muscle mass, sarcopenia, sex

## Abstract

The effects of excess aldosterone on skeletal muscle in individuals with primary aldosteronism (PA) are unknown. To examine the effects of aldosterone on skeletal muscle mass in patients with PA, by sex, 309 consecutive patients were enrolled. Skeletal muscle and fat mass of 62 patients with PA were compared with those of 247 controls with non-functioning adrenal incidentaloma (NFAI). Body composition parameters were measured using bioelectrical impedance analysis, and plasma aldosterone concentration (PAC) was measured using radioimmunoassay. The PAC in all women, but not in men, showed an inverse association with both appendicular skeletal muscle mass (ASM) (β = −0.197, *P* = 0.016) and height-adjusted ASM (HA-ASM) (β = −0.207, *P* = 0.009). HA-ASM in women (but not in men) with PA was 5.0% lower than that in women with NFAI (*P* = 0.036). Furthermore, women with PA had a lower HA-ASM than 1:1 age- and sex-matched controls with NFAI by 5.7% (*P* = 0.049) and tended to have a lower HA-ASM than 1:3 age-, sex-, and menopausal status-matched controls without adrenal incidentaloma (AI) by 7.3% (*P* = 0.053). The odds ratio (OR), per quartile increase in PAC, of low HA-ASM in women was 1.18 [95% confidence interval (CI), 1.01–1.39; *P* = 0.035]. The odds of HA-ASM in subjects with PA were 10.63-fold (95% CI: 0.83–135.50) higher, with marginal significance (*P* = 0.069) than in those with NFAI. Skeletal muscle mass in women with PA was lower than that in women with NFAI; suggesting that excess aldosterone has adverse effects on skeletal muscle metabolism.

## Introduction

Aging is associated with sarcopenia, which is characterized by loss of skeletal muscle mass and strength, and/or decline in physical performance (1). Previous studies confirm an association between sarcopenia and adverse health outcomes such as impaired cardiopulmonary performance, reduced physical capability, and increased disability and mortality (2). Asia, including Korea, is a region with a rapidly aging population; thus, sarcopenia is increasingly prevalent (3). Indeed, a national survey in Korea revealed that 11.9% of women and 12.1% of men have sarcopenia (4); therefore, it is becoming a major challenge in terms of healthy aging.

Evidence suggests that inhibiting the renin–angiotensin–aldosterone system (RAAS) may prevent the development of sarcopenia (5, 6). Indeed, the treatment of older people without congestive heart failure (CHF) with angiotensin I converting enzyme (ACE) inhibitors improves physical performance (6, 7). Aldosterone, a mineralocorticoid, is a terminal hormone of the RAAS; therefore, it may have deleterious effects on skeletal muscle (5). Aldosterone increases the loss of magnesium via the urine, thereby depleting the levels of magnesium in the muscle where it is essential for activating the Na^+^/K^+^ pumps, which regulates muscle contraction (8, 9). Aldosterone also suppresses insulin-mediated glucose uptake and increases oxidative stress in skeletal muscle (10). Furthermore, plasma aldosterone concentration (PAC) in CHF patients with cardiac cachexia is higher than that in age-matched controls without CHF (11). Blocking the mineralocorticoid receptor (MR) for aldosterone with spironolactone prevents the loss of skeletal myocytes (12), improves vascular endothelial function and muscle blood flow (13), and improves muscle contractile performance by increasing the magnesium levels and up-regulating Na^+^/K^+^ pumps (8). To date, the majority of studies examining the detrimental effects of aldosterone on skeletal muscle have been conducted in animals, in patients with CHF, or patients with alcoholic liver cirrhosis (LC), in whom muscle wasting may be caused by cachexia with impaired cardiac function or the toxic effects of alcohol. Although cachexia and sarcopenia show common pathophysiological mechanisms of underlying muscle dysfunction and muscle loss (14), there are several differences between them. Cachexia is associated with major diseases such as infections, cancer, heart disease, chronic kidney disease, chronic obstructive pulmonary disease, and stroke (15). Cachexia is weight loss caused not only by inflammatory cytokines but also by proteolytic inducers, derived from underlying diseases. On the contrary, sarcopenia is the loss of muscle mass and function, mainly associated with aging. Sarcopenia is caused by failure of satellite cell activation or by the promotion of proinflammatory cytokines (16). Therefore, it is unclear whether excess aldosterone contributes to the development of sarcopenia in the general population.

Primary aldosteronism (PA) is a disease of the adrenal gland and is characterized by levels of aldosterone that are inappropriately high for sodium status (17). Therefore, PA is a good model in which to examine the effects of excess aldosterone on human skeletal muscle. To the best of our knowledge, no study has examined skeletal muscle mass in individuals with PA. Therefore, we examined the association between PAC and skeletal muscle mass and compared the body composition of Korean patients with PA with that of those with non-functioning adrenal incidentaloma (NFAI).

## Materials and Methods

### Study Participants and Protocol

Consecutive patients (*n* = 919) with adrenal incidentaloma (AI), newly diagnosed at Asan Medical Center (AMC; Seoul, Korea) between July 2011 and December 2015, were screened (Supplementary Figure 1). Diagnosis of AI was based on the detection of an adrenal mass (size ≥1 cm) using computed tomography (CT), which was performed as part of an investigation for an unrelated disease. All patients with AI underwent biochemical evaluation to test for hormonal abnormalities. Of these, 597 patients were referred from the Health Promotion Center due to AI where they underwent bioelectrical impedance analysis (BIA); therefore, they were eligible for inclusion in this study. Two hundred and thirty eight patients with suspected hypercortisolism, pheochromocytoma, adrenal metastasis, adrenal carcinoma, adrenal tuberculosis, congenital adrenal hyperplasia, or pseudo-Cushing's syndrome were excluded. In addition, 80 patients who had taken estrogen, steroids, or thyroid hormone, or had a disorder (such as hyperthyroidism) that might affect muscle mass, were excluded. Before measuring the PAC (ng/dL) and plasma renin activity (PRA, ng/mL/h) to detect possible case of PA [determined by calculating the aldosterone to renin ratio (ARR)], all antihypertensive medications, such as angiotensin II receptor blockers ACE inhibitors, were withdrawn for ≥4 weeks to prevent possible interference with the results (17). If absolutely necessary, subjects received α-adrenergic blocker (e.g., doxazosin) and/or a non-dihydropyridine slow-release antagonist calcium channel blocker (e.g., verapamil) in accordance with recent guidelines (17). All patients were encouraged to continue with oral potassium supplementation in case of hypokalemia. And there were no restrictions on the consumption of dietary salt before testing. The subjects in the matched control group with NFAI were randomly selected from among patients who undertook a screening test via the Health Promotion Center, at AMC (Seoul, Korea) within the same periods as those in the PA group. The 57 controls were matched (1:1) to the cases according to both age (within 2.0 years) and sex.

The screening test result was considered positive if the ARR was ≥30. The diagnosis of PA was confirmed by a non-suppressed PAC value of >10 ng/dL after an intravenous saline infusion test (2 L of 0.9% saline infused over 4 h) (17). PA was excluded if the post-infusion PAC value was <5 ng/dL. The intravenous saline infusion test was repeated if the post-infusion PAC value was 5–10 ng/dL. However, PA was diagnosed without a confirmatory test in those with spontaneous hypokalemia, a PRA below the detection limits, and a PAC >20 ng/dL (17). Finally, 62 patients were diagnosed with PA (29 women and 33 men) and 247 patients were diagnosed with NFAI (76 women and 171 men) (Supplementary Figure 1). The 57 subjects in the control group with NFAI who were matched 1:1 to patients with PA in terms of sex and age (±2.0 years) were randomly selected from the 247 patients with NFAI. Furthermore, 186 controls without AI who were matched 1:3 to patients with PA in terms of sex and age (±1.0 years) were randomly selected from patients who had BIA data, which were performed in the Health Promotion Center.

Height (cm) and weight (kg) were measured (participants wore light clothing without shoes), and body mass index (BMI; kg/m^2^) was calculated. Blood pressure (BP, mmHg) was measured twice using a mercury manometer after the patient had rested for >15 min; the average value was recorded. Mean arterial pressure (MAP) was calculated as [systolic BP + (2 × diastolic BP)]/3 (mmHg). The following patient information was obtained from an interview-assisted questionnaire: regular outdoor exercise (≥30 min/d), alcohol intake (≥3 U/d), smoking habits (current smoker), previous medical or surgical procedures, history of medication use, and reproductive status (including menstruation).

The study was approved by the Institutional Review Boards at AMC, and all participants provided written informed consent.

### Bioelectrical Impedance Analysis (BIA)

Body composition was measured using a direct segmental multi-frequency BIA (In-Body 720; Biospace Co., Ltd., Seoul, Korea) apparatus. The In-Body 720 automatically estimates the weight, body mass index (BMI, kg/m^2^), fat mass (FM, kg), percent fat mass (pFM, %), and skeletal muscle mass in the arms and legs. The pFM is the ratio of FM to total body weight. Lean mass (LM, kg), is the total muscle mass. Appendicular skeletal muscle mass (ASM, kg) was calculated as the summed skeletal muscle mass in the arms and legs. Upper limb ASM (UL-ASM, kg) was calculated as the summed skeletal muscle mass in both arms, and lower limb ASM (LL-ASM, kg) was calculated as the summed skeletal muscle mass in both legs. As suggested by the Consensus Report of the Asian Working Group for Sarcopenia (1), height-adjusted ASM (HA-ASM, kg/m^2^) was defined as ASM divided by height in meters squared (ASM/height^2^) (1). Low skeletal muscle mass was defined in terms of HA-ASM using a cutoff point of <6.75 kg/m^2^ for men and <5.07 kg/m^2^ for women.

### Measurement of Hormone Levels and Biochemical Parameters

Morning blood samples were drawn after an overnight fast. The PAC and PRA were measured by radioimmunoassay (SPAC-S aldosterone and PRA kits, respectively; TFB Inc., Tokyo, Japan) using a Cobra II Gamma Counter (Packard Instrument Co., Meriden, CT). For the PAC assay, the lower limit of detection was >1.53 ng/dL, and the intra-assay and inter-assay coefficients of variation (CVs) were <3.2 and <6.7%, respectively. For the PRA assay, the lower limit of detection was >0.09 ng/mL/h and the intra-assay and inter-assay CVs were <8.3 and <9.7%, respectively.

Serum potassium levels were measured using a Roche ISE Standard Low/High (Roche Diagnostics, Mannheim, Germany) ion selective electrode (ISE) and a Cobas 8000 ISE analyzer (Roche Diagnostics). The intra-assay and inter-assay CVs were 0.5 and 1.6%, respectively. Serum creatinine was measured in a kinetic colorimetric assay using the Roche CREAJ2 kit (Roche Diagnostics) and a Cobas c702 module (Roche Diagnostics). The intra-assay and inter-assay CVs were <2.3 and <2.7%, respectively. Glomerular filtration rate (GFR) was estimated using the Cockcroft–Gault equation (18).

### Statistical Analysis

Data are expressed as the mean ± standard deviations (SD), the median (interquartile range), or number (percentage) unless stated otherwise. Baseline characteristics were compared using Student's *t*-test or the Mann–Whitney *U*-test (continuous variables) or the χ^2^ test (categorical variables). To investigate the correlation of PAC with age, we performed Pearson's correlation analysis in patients with NFAI. Interaction analysis was performed to test whether the association between PAC (presented as a continuous variable) and parameters of body composition was modified by sex (coded as 0 and 1 for women and men, respectively, and expressed as a categorical variable). The association between PAC and ASM, UL-ASM, LL-ASM, HA-ASM, FM, and pFM was evaluated by multiple linear regression analyses after adjusting for confounding factors (age, menopausal status in women, BMI, regular outdoor exercise, alcohol intake, current smoking, MAP, K^+^ levels, and GFR). To further analyze the differences in the magnitude of the association between PAC and UL-ASM and LL-ASM, the corresponding regression coefficients were compared using a previously reported equation, which is an extension of the *t*-test with unstandardized β-coefficients and standard error (SE) (19). After women and men were assigned to four groups according to PAC quartile, the multivariable-adjusted least squares mean value (95% CIs) of HA-ASM was calculated with respect to PAC quartile; these were then compared using analysis of covariance (ANCOVA) after adjusting for potential confounding factors. The multivariable-adjusted least squares mean values (95% CIs) for ASM, HA-ASM, UL-ASM, LL-ASM, FM, and pFM based on the absence/presence of PA were calculated and then compared using ANCOVA, after adjusting for potential confounding factors. The multivariable-adjusted least squares mean values (95% CIs) for HA-ASM and LM based on the absence/presence of PA were calculated and then compared with PA cases after adjusting for potential confounding factors, with the 1:1 age- and sex-matched controls with NFAI or 1:3 age-, sex-, and menopausal status-matched controls without AI, using ANCOVA. Multiple logistic regression analyses was performed to calculate the odds ratio (OR) and 95% CIs for an association between low skeletal muscle mass per increase in PAC or the presence of PA (after adjusting for potential confounders). A receiver operating characteristics (ROC) curve was constructed and the area under the curve (AUC) was measured to assess the ability of PAC to predict low skeletal muscle mass. All statistical analyses were performed using SPSS, version 22.0 (SPSS Inc., Chicago, IL). A *P* value <0.05 was deemed statistically significant.

## Results

The 309 participants enrolled in the study were categorized according to the PA status. The baseline characteristics are presented in Table 1. Women with PA (*n* = 29) tended to be younger than women with NFAI (*n* = 76; *P* = 0.061). There was no significant difference in age, menopausal status of women, and in height, weight, and GFR in both sexes, between the PA group and the NFAI group. As expected, both women and men with PA had higher systolic blood pressure (systolic BP), MAP, PAC, and ARR; and lower K^+^ levels and PRA values, than women with NFAI. PAC was negatively correlated with age (γ = −0.162, *P* = 0.001 for men and γ = −0.209, *P* = 0.001 for women) in patients with NFAI (data not shown). The baseline body composition differed by sex. The pFM was significantly higher in women (32.2 ± 6.4%) than men (23.0 ± 5.1, *P* <0.001). LM was significantly higher in men (53.5 ± 6.3 kg) than women (38.5 ± 3.9, *P* <0.001). ASM also showed the same tendency as LM (16.1 ± 2.4 kg for women vs. 23.7 ± 3.3 kg for men, *P* <0.001) (data not shown).

**Table 1:** Baseline characteristics of the study participants (*n* = 309).

**Variables**	**Women (*****n*** **= 105)**			**Men (*****n*** **= 204)**		
	**NFAI (*****n*** **= 76)**	**PA (*****n*** **= 29)**	***P***	**NFAI (*****n*** **= 171)**	**PA (*****n*** **= 33)**	***P***
Age (*y*)	54.6 ± 7.8	57.9 ± 8.3	0.061	55.2 ± 7.8	57.5 ± 6.0	0.106
Postmenopausal, *n* (%)	59 (77.6%)	24 (82.8%)	0.564	–	–	–
Height (cm)	158.4 ± 5.2	157.6 ± 5.0	0.446	169.7 ± 6.7	170.4 ± 5.1	0.574
Weight (kg)	61.4 ± 9.1	60.5 ± 10.0	0.674	74.7 ± 10.0	77.8 ± 10.8	0.116
BMI (kg/m^2^)	25.2 ± 7.1	24.3 ± 3.3	0.535	26.0 ± 2.8	26.9 ± 2.5	0.084
Systolic BP (mmHg)	**123.4 ± 13.6**	**134.0 ± 16.0**	**0.001**	**126.0 ± 11.7**	**142.3 ± 15.5**	** <0.001**
Diastolic BP (mmHg)	76.4 ± 8.6	79.2 ± 10.2	0.167	**79.6 ± 8.6**	**87.1 ± 10.3**	** <0.001**
MAP (mmHg)	**92.1 ± 9.6**	**97.4 ± 10.4**	**0.014**	**95.1 ± 8.9**	**105.5 ± 1.8**	** <0.001**
Current smoker, n (%)	3 (3.9%)	0 (0.0%)	0.278	58 (33.9%)	7 (21.2%)	0.152
Alcohol intake ≥3 U/day, *n* (%)	3 (5.1%)	0 (0.0%)	0.242	**16 (12.3%)**	**9 (32.1%)**	**0.009**
Regular exercise ≥30 min/day, *n* (%)	19 (25.0%)	2 (6.9%)	0.054	61 (35.7%)	5 (15.2%)	0.210
GFR (mL/min)	94.2 ± 27.1	92.9 ± 39.2	0.870	95.5 ± 22.0	98.0 ± 17.8	0.536
K^+^ (mEq/L)	**4.1 ± 0.3**	**3.9 ± 0.5**	**0.014**	**4.3 ± 0.3**	**4.1 ± 0.4**	**0.005**
PAC (ng/dL)	**13.1 ± 8.6**	**26.0 ± 9.5**	** <0.001**	**11.8 ± 7.0**	**23.7 ± 8.9**	** <0.001**
PRA (ng/mL/h)	**1.1 ± 0.9**	**0.5 ± 0.9**	**0.008**	**2.7 ± 4.2**	**0.3 ± 0.2**	** <0.001**
ARR ([ng/dL]/[ng/mL/h])	**29.0 ± 36.6**	**97.6 ± 61.8**	** <0.001**	**18.6 ± 33.8**	**100.3 ± 68.7**	** <0.001**

*Data are expressed as the mean ± standard deviation or as the median (interquartile range), unless indicated otherwise. Bold numbers indicate statistically significant values. NFAI, non-functioning adrenal incidentaloma; PA, primary aldosteronism; BMI, body mass index; BP, blood pressure; MAP, mean arterial pressure; GFR, glomerular filtration rate; PAC, plasma aldosterone concentration; PRA, plasma renin activity; ARR, aldosterone to renin ratio*.

Next, we tested whether any relationship existing between PAC and body composition is affected by sex. The results showed sex effects for ASM, UL-ASM, LL-ASM, and HA-ASM (*P* value for interaction = 0.008–0.030). Therefore, data for men and women were analyzed separately.

The results of the multiple linear regression analyses performed to identify any independent associations between PAC and ASM, UL-ASM, LL-ASM, HA-ASM, FM, and pFM are presented in Table 2. For women, a higher PAC was significantly associated with lower ASM, UL-ASM, LL-ASM, and HA-ASM, but not with FM and pFM, after adjusting for confounders. Despite a lack of statistical significance (*P* = 0.201), the magnitude of the inverse association between PAC and LL-ASM (β = −0.032) was larger than that between PAC and UL-ASM (β = −0.013). There was no statistically significant association between PAC and ASM, UL-ASM, LL-ASM, HA-ASM, FM, and pFM in men. Furthermore, there was no statistically significant association between PRA or ARR and ASM, UL-ASM, LL-ASM, and HA-ASM in men or women (data not shown).

**Table 2:** Multiple linear regression analysis of the association between plasma aldosterone concentration (PAC) and ASM, UL-ASM, LL-ASM, HA-ASM, FM, and pFM (*n* = 309).

**Variable**	**Women (*****n*** **= 105)**				**Men (*****n*** **= 204)**			
	**β**	**SE**	***β***	***P***	**β**	**SE**	***β***	***P***
ASM (kg)	**−0.045**	**0.018**	**−0.197**	**0.016**	0.028	0.029	0.070	0.343
UL-ASM (kg)	**−0.013**	**0.005**	**−0.197**	**0.012**	0.006	0.008	0.052	0.431
LL-ASM (kg)	**−0.032**	**0.014**	**−0.189**	**0.025**	0.022	0.022	0.074	0.338
HA-ASM (kg/m^2^)	**−0.015**	**0.006**	**−0.207**	**0.009**	−0.006	0.006	−0.060	0.341
FM (kg)	−0.047	0.026	−0.084	0.077	−0.011	0.031	−0.017	0.717
pFM (%)	−0.012	0.044	−0.022	0.783	−0.035	0.039	−0.058	0.371

*P values were calculated by multiple linear regression analysis of the PAC, adjusted for age, menopausal status (in women), body mass index, regular outdoor exercise, alcohol intake, current smoking, mean arterial pressure, glomerular filtration rate, and K^+^ level. Significant results (P < 0.05) are shown in bold*.

*ASM, appendicular skeletal muscle mass; UL-ASM, upper limb ASM; LL-ASM, lower limb ASM; HA-ASM, height-adjusted ASM; FM, fat mass; pFM, percent FM; β, unstandardized regression coefficient; SE, standard error; β, standardized regression coefficient*;

*HA-ASM: height-adjusted ASM [HA-ASM (kg/m^2^)], defined as ASM divided by body height in meters squared (ASM/height^2^)*.

**Figure 1:**
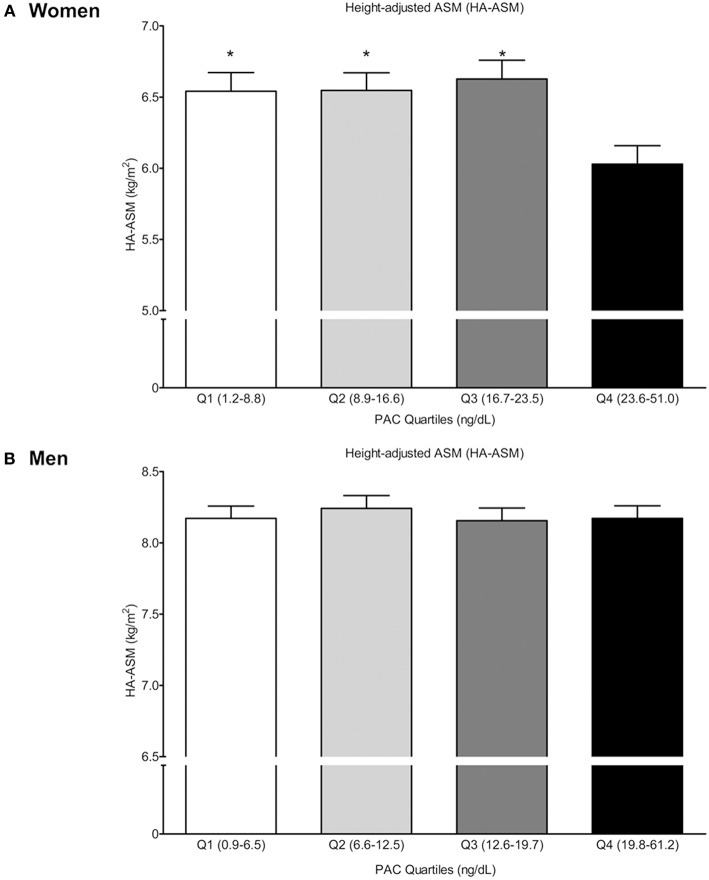
Height-adjusted ASM (HA-ASM) according to plasma aldosterone concentration (PAC) quartile. **(A)** is for women and **(B)** is for men. Values represent estimated means, with 95% confidence intervals calculated from the analysis of covariance (ANCOVA) after adjusting for age, menopausal status in women, body mass index, regular outdoor exercise, alcohol intake, current smoking, mean arterial pressure, glomerular filtration rate, and K^+^ levels. ^*^Significantly difference occurred with the highest quartile (Q4) (ANCOVA with *post-hoc* analysis).

After adjusting for potential confounders, estimation of the multivariable-adjusted least squares mean HA-ASM according to PAC quartiles (Figure 1) revealed that women in the highest quartile (quartile 4: 23.6–51.0 ng/dL) had a lower HA-ASM than those in the other quartiles (quartiles 1–3: 1.2–23.5 ng/dL); specifically, the values were 7.8% lower than those in quartile 1 (*P* = 0.007), 7.9% lower than those in quartile 2 (*P* = 0.005), and 9.0% lower than those in quartile 3 (*P* = 0.002) (Figure 1A). There was no significant difference in HA-ASM between PAC quartiles 1, 2, and 3 (*P* = 0.660–0.974). For men, the association between PAC and HA-ASM did not show a threshold effect (Figure 1B).

**Figure 2:**
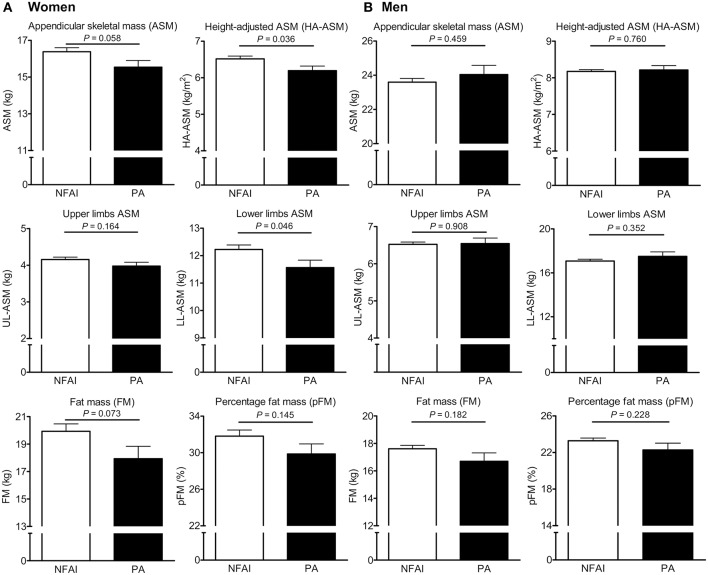
Differences in appendicular skeletal muscle mass (ASM), height-adjusted ASM (HA-ASM), upper limb ASM (UL-ASM), lower limb ASM (LL-ASM), fat mass (FM), and percent FM (pFM) between subjects with and without primary aldosteronism (PA). **(A)** is for women and **(B)** is for men. Values represent estimated means, with 95% confidence intervals calculated from analysis of covariance (ANCOVA) after adjusting for age, menopausal status in women, body mass index, regular outdoor exercise, alcohol intake, current smoking, mean arterial pressure, glomerular filtration rate (GFR), and K^+^ levels. NFAI, non-functioning adrenal incidentaloma.

Next, we used ANCOVA to estimate differences in ASM, HA-ASM, UL-ASM, LL-ASM, FM, and pFM between participants with PA and NFAI (after adjusting for all potential confounders) (Figure 2). For women with PA, LL-ASM was 5.4% lower (*P* = 0.046) and HA-ASM was 4.9% lower (*P* = 0.036) than those for women without PA (Figure 2A). There was no difference in UL-ASM. For men, there was no statistically significant difference in ASM, HA-ASM, UL-ASM, LL-ASM, FM, and pFM between the PA and NFAI groups (Figure 2B). We compared HA-ASM and LM between patients with PA (*n* = 57) and 1:1 age- (±2.0 years), and sex- matched controls with NFAI (*n* = 57) (Supplementary Table 1, Supplementary Figure 2). As shown in Supplementary Figure 2; 24 women with PA had lower HA-ASM than 1:1 age- and sex-matched 24 women with NFAI controls by 5.7% (*P* = 0.049) after adjusting for all potential confounders. For men, there was no statistically significant difference in HA-ASM and LM between the PA (*n* = 33) and 1:1 age-, and sex- matched controls with NFAI (*n* = 33). We also compared HA-ASM and LM between patients with PA (*n* = 62) and 1:3 sex-, age- (±1.0 years), and menopausal status-matched controls without AI (*n* = 186) (Supplementary Table 2, Supplementary Figure 2). Women with PA (*n* = 29) tended to have lower HA-ASM than 1:3 age-, sex-, and menopausal status-matched controls women without AI (*n* = 87) by 7.3% (*P* = 0.054). For men, there was no statistically significant difference in HA-ASM and LM between the PA (*n* = 33) and control groups (*n* = 99).

Finally, we performed multiple logistic regression analyses to identify any association between PAC or the presence of PA and the risk of lower skeletal muscle mass (Table 3). For women, the odds ratio (OR) [95% confidence interval (95% CIs)] per quartile increase in PAC for lower skeletal muscle mass was 1.18 (1.01–1.39). In addition, the OR of the association between PA and lower skeletal muscle mass in women was 10.63-fold higher (95% CI, 0.83–135.50) than that for the association between NFAI and lower skeletal muscle mass (Table 3). A ROC curve analysis performed to determine the PAC threshold for predicting low skeletal muscle mass in women revealed an AUC of 0.734 (95% CI, 0.639–0.875) (Supplementary Figure 3). The cutoff value, which corresponded to Youden's index (20), was 29 ng/dL. A PAC value ≥29.0 ng/dL predicted low skeletal muscle mass with a sensitivity of 57.1% and a specificity of 92.9%. For women, the OR (95% CI) of the association between PAC values ≥29.0 ng/dL and low skeletal muscle mass was 139.17 (2.40–8069.74).

**Table 3:** Multiple logistic regression analyses to determine the odds ratio (OR) and 95% confidence intervals (95% CIs) for the association between lower skeletal muscle mass^*^ and plasma aldosterone concentration (PAC) or primary aldosteronism (PA).

	**Women**		**Men**	
	**OR (95% CI)**	***P***	**OR (95% CI)**	***P***
PAC	**1.18 (1.01−1.39)**	**0.035**	1.05 (0.94–1.17)	0.427
PA	10.63 (0.83–135.50)	0.069	2.96 (0.27–32.68)	0.376

*Multivariate analysis was adjusted for age, menopausal status in women, body mass index, current smoking, alcohol intake, regular outdoor exercise, mean arterial pressure, glomerular filtration rate, and K^+^ levels. Significant results (P < 0.05) are shown in bold*.

*Height-adjusted appendicular skeletal muscle mass [HA-ASM (kg/m^2^)] was defined as ASM divided by body height in meters squared (ASM/height^2^)*.

* *Lower skeletal muscle mass was defined according to height-adjusted ASM (HA-ASM) using a cutoff of <6.75 kg/m^2^ for men and <5.07 kg/m^2^ for women (1)*.

## Discussion

The data presented herein reveal an inverse association between PAC and ASM, UL-ASM, LL-ASM, and HA-ASM (after adjusting for potential confounders) in women. This was not the case for men. Consistent with this, LL-ASM and HA-ASM in women (but not in men) with PA were lower than in women with NFAI. Furthermore, women with PA had lower HA-ASM than 1:1 age- and sex-matched controls with NFAI, and tended to have lower HA-ASM than 1:3 age-, sex-, and menopausal status-matched controls without AI. The odds of low skeletal muscle mass were higher according to the PAC and high PAC level in women, but not in men. To the best of our knowledge, this study presents the first clinical evidence that excess aldosterone might contribute to a reduction in skeletal muscle mass, particularly in women.

Despite the lack of a statistically significant association between PRA and parameters indicative of skeletal muscle mass, the finding of an inverse association between PAC and ASM, UL-ASM, LL-ASM, or HA-ASM suggests that excess aldosterone *per se* has a detrimental effect on skeletal muscle mass. Although aldosterone increases Na^+^/K^+^ pumps activity in skeletal muscle of patients with Conn's syndrome (21), our data reported that aldosterone has deleterious effects on skeletal muscle mass in humans without CHF. Furthermore, this finding is in agreement with those reported in an animal study showing that injecting rats with aldosterone induces apoptosis of myocytes in skeletal muscle (12). Women in the highest PAC quartile (PAC ≥23.6 ng/dL) had a lower HA-ASM than those in the other three quartiles. Also, PAC ≥29.0 ng/dL was associated with low skeletal muscle mass in women. These findings agree with those reported in another study showing that PAC in CHF patients with cachexia was 2-fold higher than that in non-cachectic CHF patients, and more than 3-fold higher than that in age-matched individuals (35.5 ng/dL vs. 18.0 ng/dL and 10.8 ng/dL, respectively), despite the possibility that impaired cardiac function was a confounding factor (11). Taken together, the results of both the previous and present studies suggest the detrimental effects of aldosterone excess on the skeletal muscle mass in subjects with a high PAC. Indeed, several studies suggest that spironolactone prevents the loss of skeletal myocytes in animals (12), improves vascular endothelial function and muscle blood flow in patients with CHF (13), and improves muscle contractile performance by increasing magnesium levels and by up-regulating Na^+^/K^+^ pumps in skeletal muscle of patients with alcoholic LC (8). However, these patients may have experienced muscle wasting due to cachexia from impaired cardiac function or the toxic effects of alcohol. Therefore, our study excluded the combined effects of underlying disease, reported in previous studies and identified the effects of aldosterone excess *per se* on the development of sarcopenia in the general population.

A previous study showed that subjects with NFAI may have a higher risk of atherosclerosis than age-, or sex-matched subjects without adrenal gland lesions, and suggested that the body composition of patients with NFAI may differ from that of subjects without AI (22). Therefore, we compared the muscle mass of PA patients with 1:3 age-, sex-, and menopausal status-matched controls without AI. And we found that HA-ASM in women, but not in men, was lower in patients with PA than in age-, sex-, and menopausal status-matched controls without AI, although with a marginal significance. Therefore, these results also suggest a detrimental effect of PA on skeletal muscle metabolism in humans.

Another interesting finding reported herein is that the deleterious effects of excess aldosterone on skeletal muscle mass occurred only in women, and that it was more evident in the lower limbs than in the upper limbs. The reason for this sex dimorphism is unknown; differences in 11β-hydroxysteroid dehydrogenase (11β-HSD) expression and in PAC according to sex might be subsidiary reasons. First, we speculate about the sexual dimorphism of 11β-HSD. In the skeletal muscle, there are two isoforms of 11β-HSD; 11β-HSD1 (converting inactive cortisone to active cortisol), and 11β-HSD2 (converting cortisol to cortisone), resulting in the protection of MR from cortisol and the regulation of the binding of aldosterone to MR (23, 24). Upregulation of skeletal muscle 11β-HSD1 occurring with age in women, but not in men (24) might act as a local tissue amplifier of cortisol, mimicking aldosterone as an MR agonist, due to the high affinity of cortisol equivalent to aldosterone for MR (25, 26). Second, the 12/106 women (11.3%) and 6/204 men (2.9%), *P* = 0.003, with high PAC (≥29.0 ng/dL), may be more vulnerable to the deleterious effects of excess aldosterone. ASM in women with PA was 5.4% lower in the lower limbs (*P* = 0.046), but only 4.3% lower in the upper limbs (*P* = 0.164), than that in women without PA. This pattern of greater muscle weakness in the lower limbs than the upper limbs in those with PA is similar to that observed in patients with overt hypercortisolism; thus, the issue remains unresolved (27).

Although it is assumed that old age starts at about 65 years of age, several studies showed that age-dependent loss of skeletal muscle mass starts in middle-aged adults between 45 and 65 years of age (28, 29). In line with the results of a previous study, decreased HA-ASM in Korean men accelerated after 40 years of age, and that in Korean women began after around 55 years in a study on the assessment of muscle mass in Koreans, using the Korea National Health and Nutrition Examination Survey IV (30).Therefore, our study showed the effects of aldosterone excess on skeletal muscle mass in the early phase of aging-related loss of skeletal muscle mass. Generally, the secretory functions of hormones fall with aging. In line with the results of previous studies showing that PAC decreases with age (31, 32), we also showed the inverse association of PAC with age. However, these results could not exclude the inappropriate activation of MR, as well as the efficacy of pharmacological MR antagonism therapy in aging populations (31). Since there is no report about the role of aldosterone/MR on skeletal muscle function in aging-related skeletal muscle mass loss in humans, further studies in those aged over 65 years and long-term follow-up period will be needed.

A major strength of this study is that we minimized selection bias by screening consecutive subjects with newly diagnosed AI. Also, we analyzed patients with PA as an ideal human model to explain the effect of excess aldosterone on skeletal muscle. However, the study has several limitations. First, the accuracy of BIA readings is affected by variable parameters such as body temperature, position, and hydration status (1, 33). However, because BIA is reproducible, inexpensive, and easy to use, and it has been validated for sarcopenia diagnosis (1, 33), the Asian Working Group for Sarcopenia regards the method as suitable for measuring muscle mass (1). Second, we did not measure physical performance or muscle strength. When diagnosing sarcopenia, it is essential to identify reductions in muscle function (physical function or muscular strength), not only muscle mass (1, 33). Neither physical performance nor muscle strength was measured in our cohort; we analyzed only the lower skeletal muscle mass in patients with PA. Therefore, other parameters including physical performance or muscle strength should be measured to enforce our findings of the hazard effect of muscle loss in patients with PA in future studies.

In summary, women with PA had lower skeletal muscle mass than those with NFAI, suggesting that excess aldosterone has an adverse effect on skeletal muscle. Further studies are required to identify the complex mechanisms underlying the marked increase in aldosterone concentration and sarcopenia in aging humans.

## Data Availability

All datasets generated for this study are included in the manuscript and/or the Supplementary Files.

## Author Contributions

JHK and SHL contributed equally to this study. JHK and SHL had full access to all of the data in the study and take responsibility for the integrity of the data and the accuracy of the data analysis. MKK, S-EL, YYC, and SS conception or design of the work. MKK and S-EL analysis or interpretation of data for the work. B-JK, K-HS, and J-MK acquisition of data for the work. JHK, SHL, B-JK, K-HS, and J-MK drafting of the work or revising it critically for important intellectual content. MKK, S-EL, YYC, SS, B-JK, K-HS, JHK, J-MK, and SHL final approval of the version to be published.

### Conflict of Interest Statement

The authors declare that the research was conducted in the absence of any commercial or financial relationships that could be construed as a potential conflict of interest.
