# Supplementary material for: The Differential Effect of Excess Aldosterone on Skeletal Muscle Mass by Sex
Source: Front Endocrinol (Lausanne). 2019 Mar 29;10:195. doi: 10.3389/fendo.2019.00195 (PMC6450066; doi:10.3389/fendo.2019.00195)
Supplement: Supplementary file 1 [file Data_Sheet_1.docx]

Supplementary Material

The Differential Effect of Excess Aldosterone on Skeletal Muscle Mass by Sex

Mi Kyung Kwak^1^, Seung-Eun Lee^2^, Yoon Young Cho^3^, Sunghwan Suh^4^, Beom-Jun Kim^1^, Kee-Ho Song^5^, Jung-Min Koh^1^, Jae Hyeon Kim^2,*^, and Seung Hun Lee^1,*^

*** Correspondence:**

Jae Hyeon Kim

[jaehyeonkim26@gmail.com](mailto:jaehyeonkim26@gmail.com).

Seung Hun Lee

[hun0108@amc.seoul.kr](mailto:hun0108@amc.seoul.kr).

**Supplementary Figure 1.** Flow diagram showing the selection of the study population


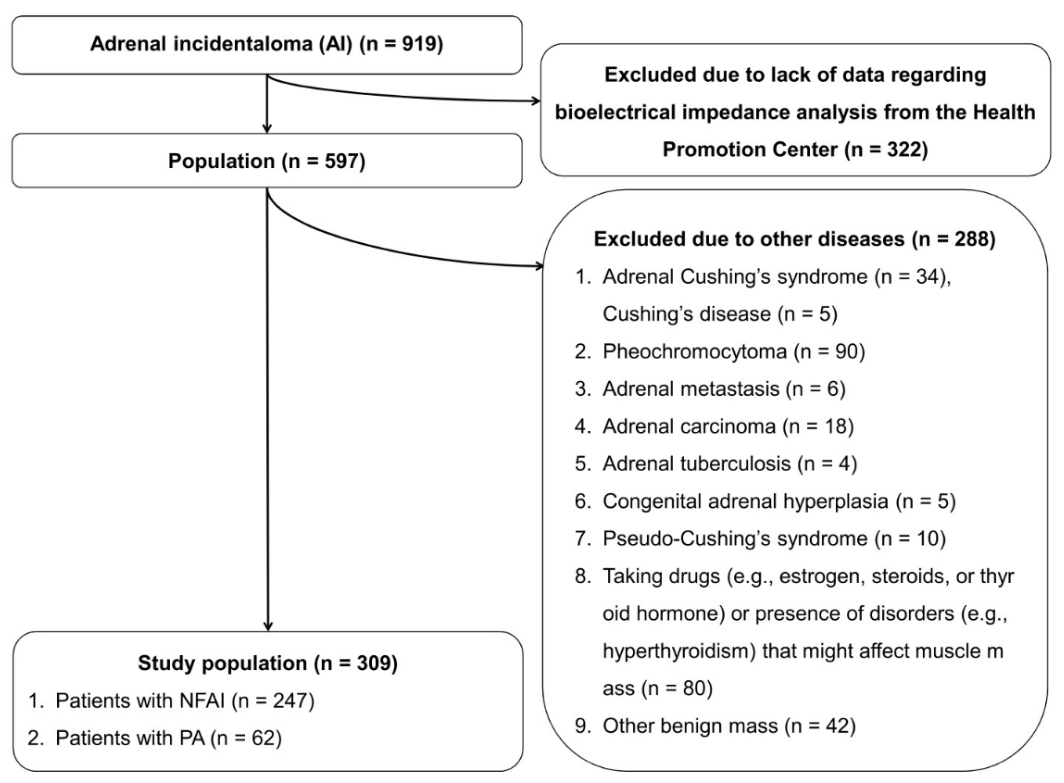


NFAI**,** non-functioning adrenal incidentaloma; PA, primary aldosteronism

**Supplementary Figure 2.** Differences in height-adjusted appendicular skeletal muscle mass (HA-ASM) and lean mass (LM) between patients with primary aldosteronism (PA) and 1:1 age- and sex- matched controls with non-functioning adrenal incidentaloma (NFAI) or 1:3 age-, sex-, and menopause status- matched controls without adrenal incidentaloma (AI)

**
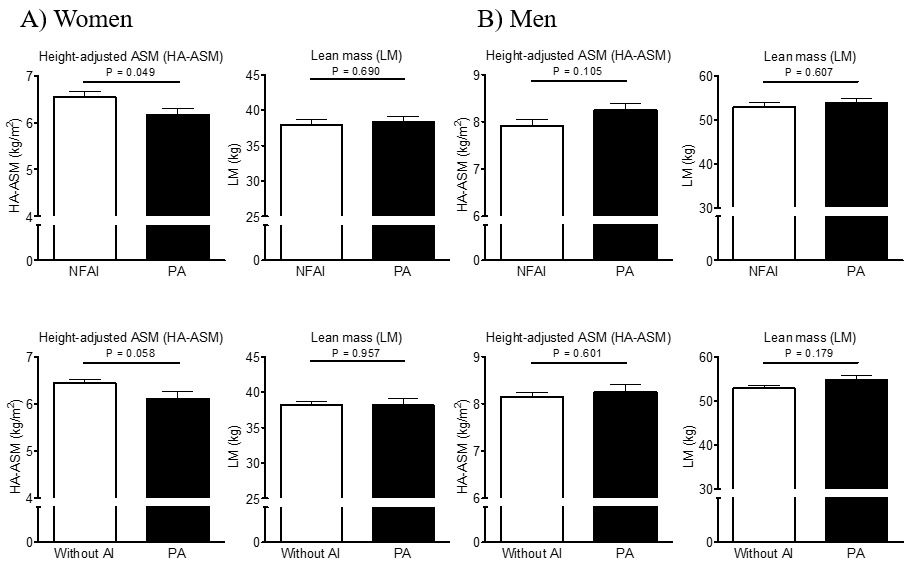
**

Values represent estimated means, with 95% confidence intervals calculated from analysis of covariance (ANCOVA) after adjusting for age, menopausal status in women, body mass index, regular outdoor exercise, alcohol intake, current smoking, mean arterial pressure, glomerular filtration rate (GFR), and K^+^ levels.

**Supplementary Figure 3.** A receiver operating characteristics (ROC) curve analysis to determine the plasma aldosterone concentration (PAC) threshold for predicting low skeletal muscle mass in women


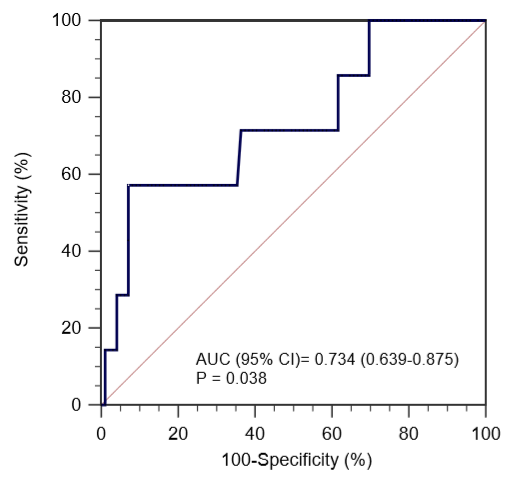


**Supplementary Table 1.** Baseline characteristics of 57 patients with primary aldosteronism (PA) and 1:1 age- and sex- matched 57 controls with non-functioning adrenal incidentaloma (NFAI) (n = 114)

| Variables | Women (n = 48) | | | Men (n = 66) | | | |
| --- | --- | --- | --- | --- | --- | --- | --- |
|  | NFAI (n = 24) | PA (n = 24) | P |  | NFAI (n = 33) | PA (n = 33) | P |
| Age (y) | 56.7 ± 8.0 | 56.3 ± 7.9 | 0.886 |  | 57.6 ± 6.1 | 57.5 ± 6.0 | 0.951 |
| Postmenopausal, n (%) | 20 (83.3%) | 19 (79.2%) | 0.999 |  | – | – | – |
| Height (cm) | 157.5 ± 5.0 | 158.2 ± 4.9 | 0.646 |  | 168.6 ± 6.4 | 170.4 ± 5.1 | 0.200 |
| Weight (kg) | 59.5 ± 9.2 | 61.6 ± 10.6 | 0.466 |  | **71.9 ± 9.6** | **77.8 ± 10.8** | **0.022** |
| BMI (kg/m^2^) | 24.0 ± 3.8 | 24.6 ± 3.5 | 0.575 |  | **25.3 ± 2.8** | **26.7 ± 2.6** | **0.035** |
| Systolic BP (mmHg) | 128.5 ± 16.2 | 132.8 ± 14.4 | 0.328 |  | **125.3 ± 9.9** | **142.3 ± 15.5** | **<0.001** |
| Diastolic BP (mmHg) | 78.8 ± 11.2 | 79.7 ± 10.0 | 0.766 |  | **78.0 ± 9.5** | **87.1 ± 10.3** | **<0.001** |
| MAP (mmHg) | 95.1 ± 12.4 | 97.4 ± 9.9 | 0.493 |  | **93.7 ± 8.6** | **105.5 ± 10.6** | **<0.001** |
| Current smoker, n (%) | 1 (4.2%) | 0 (0.0%) | 0.999 |  | 15 (45.5%) | 7 (21.2%) | 0.068 |
| Alcohol intake ≥3 U/day, n (%) | 0 (0.0%) | 0 (0.0%) | 0.999 |  | 5 (15.2%) | 9 (27.3%) | 0.366 |
| Regular exercise ≥30 min/day, n (%) | **5 (20.8%)** | **1 (4.2%)** | **0.019** |  | **17 (51.5%)** | **5 (15.2%)** | **0.004** |
| GFR (mL/min) | 90.7 ± 19.3 | 98.0 ± 17.8 | 0.114 |  | 91.1 ± 24.5 | 96.8 ± 42.0 | 0.567 |
| K^+^ (mEq/L) | 4.2 ± 0.3 | 4.1 ± 0.4 | 0.242 |  | 4.2 ± 0.4 | 3.9 ± 0.5 | 0.046 |
| PAC (ng/dL) | **14.5 ± 7.3** | **26.1 ± 9.5** | **<0.001** |  | **14.6 ± 6.6** | **23.7 ± 8.9** | **<0.001** |
| PRA (ng/mL/h) | 0.8 ± 0.8 | 0.5 ± 1.0 | 0.393 |  | **2.4 ± 4.8** | **0.3 ± 0.2** | **0.018** |
| ARR ([ng/dL]/[ng/mL/h]) | **37.4 ± 33.1** | **98.5 ± 61.7** | **<0.001** |  | **29.9 ± 54.6** | **100.3 ± 68.7** | **<0.001** |

Data are expressed as the mean ± standard deviation or as the median (interquartile range), unless indicated otherwise. **Bold** numbers indicate statistically significant values.

NFAI, non-functioning adrenal incidentaloma; PA, primary aldosteronism; BMI, body mass index; BP, blood pressure; MAP, mean arterial pressure; GFR, glomerular filtration rate; PAC, plasma aldosterone concentration; PRA, plasma renin activity; ARR, aldosterone to renin ratio.

**Supplementary Table 2.** Baseline characteristics of the 62 patients with primary aldosteronism (PA) and 1:3 age-, sex-, and menopause status- matched 186 controls without adrenal incidentaloma (AI) (n = 248)

| Variables | Women (n = 116) | | | Men (n = 132) | | | |
| --- | --- | --- | --- | --- | --- | --- | --- |
|  | Without AI (n = 87) | PA (n = 29) | P |  | Without AI (n = 99) | PA (n = 33) | P |
| Age (y) | 57.9 ± 8.2 | 57.9 ± 8.3 | 0.999 |  | 57.5 ± 6.0 | 57.5 ± 6.0 | 0.999 |
| Postmenopausal, n (%) | 71 (81.6%) | 24 (82.8%) | 0.999 |  | – | – | – |
| Height (cm) | **155.0 ± 5.7** | **157.6 ± 5.0** | **0.029** |  | **167.9 ± 6.4** | **170.4 ± 5.1** | **0.040** |
| Weight (kg) | 59.0 ± 8.8 | 60.5 ± 10.0 | 0.444 |  | **68.4 ± 10.0** | **77.8 ± 10.8** | **<0.001** |
| BMI (kg/m^2^) | 24.5 ± 2.9 | 24.3 ± 3.3 | 0.756 |  | **24.2 ± 3.0** | **26.7 ± 2.6** | **<0.001** |
| Systolic BP (mmHg) | **123.4 ± 17.4** | **134.0 ± 16.0** | **0.004** |  | **121.2 ± 16.1** | **142.3 ± 15.5** | **<0.001** |
| Diastolic BP (mmHg) | 77.1 ± 9.6 | 79.2 ± 10.2 | 0.332 |  | **78.8 ± 10.9** | **87.1 ± 10.3** | **<0.001** |
| MAP (mmHg) | **92.6 ± 11.4** | **97.4 ± 10.4** | **0.044** |  | **92.9 ± 11.8** | **105.5 ± 10.6** | **<0.001** |
| Current smoker, n (%) | 6 (7.0%) | 0 (0.0%) | 0.328 |  | **62 (62.6%)** | **7 (21.2%)** | **<0.001** |
| Alcohol intake ≥3 U/day, n (%) | 3 (3.5%) | 0 (0.0%) | 0.730 |  | 44 (44.4%) | 9 (27.3%) | 0.124 |
| Regular exercise ≥30 min/day, n (%) | 15 (17.4%) | 2 (6.9%) | 0.280 |  | 7 (7.1%) | 5 (15.2%) | 0.294 |
| GFR (mL/min) | 80.9 ± 17.5 | 92.9 ± 39.2 | 0.121 |  | **83.3 ± 19.2** | **98.0 ± 17.8** | **<0.001** |

Data are expressed as the mean ± standard deviation or as the median (interquartile range), unless indicated otherwise. **Bold** numbers indicate statistically significant values.

Without AI, without adrenal incidentaloma; PA, primary aldosteronism; BMI, body mass index; BP, blood pressure; MAP, mean arterial pressure; GFR, glomerular filtration rate.

**
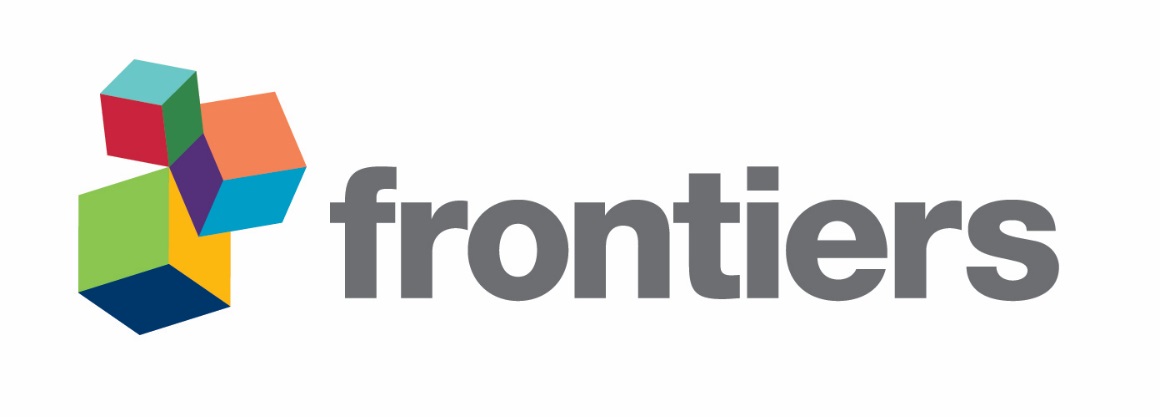
**
